# Prostate cryoablation combined with androgen deprivation therapy for newly diagnosed metastatic prostate cancer: a propensity score-based study

**DOI:** 10.1038/s41391-021-00335-2

**Published:** 2021-03-04

**Authors:** Ning wang, Yangtian Ye, Minhua Deng, Diwei Zhao, Lijuan Jiang, Dong Chen, Zhiming Wu, Yanjun Wang, ZhiYong Li, Zhenyu Yang, Jibin Li, Fangjian Zhou, Yonghong Li

**Affiliations:** 1 Department of Urology, Sun Yat-Sen University Cancer Center, Guangzhou, Guangdong China; 2 State Key Laboratory of Oncology in South China; Collaborative Innovation Cencer for Cancer Medicine, Sun Yat-Sen University Cancer Center, Guangzhou, Guangdong China; 3 Department of Urology, the First Affiliated Hospital of Jinan University, Guangzhou, China; 4 Department of Clinical Research, Sun Yat-Sen University Cancer Center, Guangzhou, Guangdong China

**Keywords:** Prostate cancer, Cancer therapy, Cancer therapy

## Abstract

**Background:**

Several studies showed that androgen deprivation therapy (ADT) plus local treatment of prostate could improve metastatic prostate cancer (mPCa) patients’ survival. To date there are few studies analyzed the value of prostate cryoablation in mPCa. The objective of our analysis is to evaluate the oncological results and clinical value of prostate cryoablation combined with ADT compared with ADT alone in newly diagnosed mPCa patients.

**Methods:**

Newly diagnosed mPCa patients undergoing cryoablation plus ADT (group A) between January 2011 and November 2018 were identified. Patients receiving ADT alone (group B) were selected from the same institutional prostate cancer database by propensity score matching based on clinical characteristics. Oncological results and clinical value in symptom control and primary lesion treatment were compared.

**Results:**

Fifty-four patients were included in each group. Prostate cryoablation was well tolerated. The median follow-up time was 40 (27–53) and 39 (31–54) months in group A and group B, respectively. Patients in group A had a lower median prostate-specific antigen (PSA) nadir (0.025 ng/mL vs. 0.230 ng/mL, *p* = 0.001), longer median failure-free survival (FFS) (39 months vs. 21 months, *p* = 0.005), and median metastatic castration-resistant prostate cancer (mCRPC)-free survival (39 months vs. 21 months, *p* = 0.007). No difference in cancer-specific survival and overall survival was found between the two groups. Multivariate Cox analysis showed combination therapy reduced the risk of FFS by 45.8% (HR = 0.542 [95% CI 0.329–0.893]; *p* = 0.016). Patients in group A had better clinical relief of urinary symptoms (79.1 vs. 59.1%, *p* = 0.044) and required less treatment of primary lesions for symptomatic relief (13.0 vs. 31.5%, *p* = 0.021).

**Conclusions:**

Prostate cryoablation plus ADT decreases PSA nadir, prolongs FFS and mCRPC-free survival, relieves urinary symptoms and reduces the need for treating primary lesions in newly diagnosed mPCa patients compared to ADT alone.

## Introduction

Prostate cancer (PCa) is the second leading cause of cancer death among men in the United States and Europe [1]. In China, the rate of PCa increased dramatically [2], and most newly diagnosed patients present metastatic prostate cancer (mPCa). The overall 5-year survival rate of PCa and mPCa is 98.2 and 30.0%, respectively [3].

Androgen deprivation therapy (ADT) is a standard treatment for newly diagnosed mPCa patients. This treatment is effective in up to 95% of patients; however, cancer progresses to metastatic castration-resistant prostate cancer (mCRPC) in most cases [4]. A randomized controlled trial [5] showed that the median failure-free survival (FFS) in mPCa patients treated with ADT was 11 months. Although drug therapies have improved significantly in recent years, mCRPC is still an intractable problem for oncologists. Furthermore, more than one-third of patients without primary lesion treatment developed ureteric obstruction, bladder outlet obstruction (BOO), and other complications because of the primary lesion progression of PCa [6].

Data from the Surveillance Epidemiology and End Result (SEER) database [7] from 2014 showed that the 5-year overall survival (OS) and cancer-specific survival (CSS) were significantly higher in mPCa patients receiving definitive treatment for PCa (cytoreductive radical prostatectomy [CRP] or brachytherapy) compared with untreated patients. Heidenreich et al. [8] showed that ADT plus CRP was feasible for patients with longer life expectancy and reduced the risk of locally recurrent PCa and local complications. The STAMPEDE study [9] indicated that ADT plus prostate radiotherapy improved FFS in mPCa patients and prolonged OS in low metastatic burden patients. The HORRAD study [10] showed that ADT combined with external beam radiation therapy improved prostate-specific antigen (PSA) recurrence-free survival in patients with primary bone mPCa.

Cryoablation (CA) is a minimally invasive and feasible local treatment for PCa. The best practice statement on prostate cryosurgery 2008 [11] reported the safety, reliability, and indications of CA for treating localized PCa. However, few studies have evaluated the outcomes of mPCa after primary lesion CA.

The purpose of this study is to evaluate the oncological results and clinical value of prostate CA plus ADT compared with ADT alone for newly diagnosed mPCa.

## Patients and methods

### Patient selection

Data were collected from an institutional review board-approved PCa database from the Sun Yat-Sen University Cancer Center. This retrospective study complied with the Declaration of Helsinki and was approved by the Ethics Committee of Sun Yat-Sen University Cancer Center. Patients gave informed written consent before they chose cryoablation and ADT as their therapy. Patients received digital rectal examination, assessment of clinical symptoms, and serum PSA level. Then, radiological examinations including isotope bone scan, computed tomography (CT) and/or magnetic resonance imaging (MRI) were performed to assess the tumor condition of the prostate and other parts of the body. Prostate biopsy and pathological analysis were then carried out. The inclusion criteria were patients who were newly diagnosed mPCa and received ADT plus whole-gland prostate CA or ADT alone as the first-line therapy. The patients who received chemotherapy, abiraterone acetate plus prednisone, radiotherapy, or prostatectomy at the mHSPC stage were excluded. Fifty-four consecutive patients underwent ADT plus whole-gland prostate CA (group A), and 112 cases received ADT alone at the metastatic hormone-sensitive prostate cancer (mHSPC) stage from January 2011 to November 2018.

### Study variables

Patient baseline data on age, urinary symptoms, ECOG performance status (ECOG PS), PSA levels, Gleason score, clinical stage, and metastatic burden were collected. Posttreatment data, including urinary symptoms, PSA levels, imaging information, complications, therapies after mCRPC, and patient survival, were recorded. mCRPC was defined according to the European Association of Urology Guideline [12] as castrate serum testosterone <50 ng/dL (1.7 nmol/L) combined with one of these events: biochemical progression (three consecutive increases in PSA at least 1 week apart resulting in two 50% increases above nadir, and PSA > 2 ng/mL), and radiological progression (appearance of new lesions, including at least two new bone lesions on bone scan or a soft tissue lesion using Response Evaluation Criteria in Solid Tumors). The metastatic burden was classified as high or low according to the criteria established in the STAMPEDE trial [9]. High metastatic burden was defined as at least four bone metastases with at least one outside pelvis or the vertebral bodies; or visceral metastases; or both. Others were seen as patients with low metastatic burden. The primary endpoint was FFS, which was defined [13] as the interval from diagnosis of PCa to one of the following: PSA > 4 ng/mL and at least 50% increase over the lowest level; progression of lymph nodes, local disease, or distant metastases; or death due to PCa. CSS was defined as the period from diagnosis of PCa to death from PCa. OS was defined as the period from the initial diagnosis of PCa to death from any cause. Urinary symptom relief at the mHSPC stage and complications due to primary lesions at the mCRPC stage were assessed by patient complaints and medical history. Urinary symptoms were defined as lower urinary tract symptoms and hematuria. Local complications due to primary lesions were defined as BOO, hematuria, acute urinary retention, and ureteric obstruction, as detailed previously^6^.

### CA and ADT

Whole-gland prostate CA was conducted under transrectal ultrasonography guidance. The process involves two freeze-thaw cycles [14]. Each patient received prostate cryosurgery at the mHSPC stage. ADT included the continuous infusion of a luteinizing hormone-releasing hormone agonist or bilateral orchiectomy, and serum testosterone was measured to guarantee that the enrolled patients presented the minimum accepted level (<50 ng/dL).

### Follow-up

After treatment, all patients were followed-up every 1–3 months. Digital rectal examination and assessment of clinical symptoms and serum PSA levels were performed at 1- to 3-month intervals. Radiological examinations, including ﻿isotope bone scan, CT, or MRI, were performed every 6–12 months. In patients with PSA progression or clinical symptoms, radiological examination was carried out to assess disease activity and formulate a treatment plan.

### Statistical analysis

To compare prostate CA plus ADT vs. ADT alone, baseline clinical characteristics, including age, ECOG PS, PSA levels, Gleason score, clinical stage, and metastatic burden, were balanced using propensity score matching (PSM) at a ratio of 1:1. Log-rank calculation formula of sample size was applied to calculate sample power. In the descriptive analysis, normally distributed quantitative data were shown as means and standard deviations, and non-normally distributed data were expressed as median and interquartile range. Qualitative data were represented as frequencies and percentages. A two-sample *t*-test was used to compare the means of continuous variables with normal distribution, and the Wilcoxon rank sum test was used to compare non-normally distributed continuous variables. A Chi-square test was used for categorical data. The Kaplan–Meier method was used for survival analysis, and the log-rank test was used for statistical significance testing. Univariate and multivariate Cox proportional hazards analyses were performed to assess the prognostic value of additional CA. A two-sided *p* value of less than 0.05 was considered to indicate a statistically significant difference. PSM and statistical analyses were performed using SPSS software version 25.0.

## Results

### Patient characteristics

A total of 108 patients were evaluated after PSM (54 in group A [CA plus ADT] and 54 in group B [ADT only]). The baseline characteristics of the study population are shown in Table 1. Seven (13.0%) patients in group A had lung metastases. Eight (14.8%) patients in group B had metastasis to different organs, including lungs (*N* = 5), meninges (*N* = 1), lung and thyroid (*N* = 1), and lung and liver (*N* = 1).

**Table 1:** Baseline characteristics of the study population after propensity score matching.

Variable	Total cohort	Group A	Group B	*p* value
Patients (*n*)	108	54	54	
Age (years)	67.8 ± 8.7	68.2 ± 8.3	67.5 ± 9.2	0.685
ECOG PS				0.428
0	91 (84.3%)	44 (81.5%)	47 (87.0%)	
>0	17 (15.7%)	10 (18.5%)	7 (13.0%)	
PSA (ng/mL)	78.8 (32.9–156.2)	63.1 (31.5–116.8)	97.2 (35.6–217.6)	0.144
Gleason score				0.326
≥8	65 (60.2%)	30 (55.6%)	35 (64.8%)	
<8	43 (39.8%)	24 (44.4%)	19 (35.2%)	
T Stage				0.555
≥3	65 (60.2%)	31 (57.4%)	34 (63.0%)	
<3	43 (39.8%)	23 (42.6%)	20 (37.0%)	
N Stage				0.123
0	50 (46.3%)	29(53.7%)	21 (38.9%)	
1	58 (53.7%)	25 (46.3%)	33 (61.1%)	
Visceral metastases				0.781
0	93 (86.1%)	47 (87.0%)	46 (85.2%)	
1	15 (13.9%)	7 (13.0%)	8 (14.8%)	
Metastatic burden				0.847
Low	49 (45.4%)	25 (46.3%)	24 (44.4%)	
High^a^	59 (54.6%)	29 (53.7%)	30 (55.6%)	
Prostate volume (mL^3^)	30.6 (19.5–43.4)	31.3 (20.7–36.8)	30.4 (18.9–49.0)	0.351

Group A, cryoablation combined with androgen deprivation therapy (ADT); group B, ADT alone.

*ECOG PS* Eastern Cooperative Oncology Group Performance Status, *PSA* prostate-specific antigen.

^a^at least four bone metastases with at least one outside pelvis or the vertebral bodies; or visceral metastases; or both.

### Oncological outcomes

**Fig. 1:**
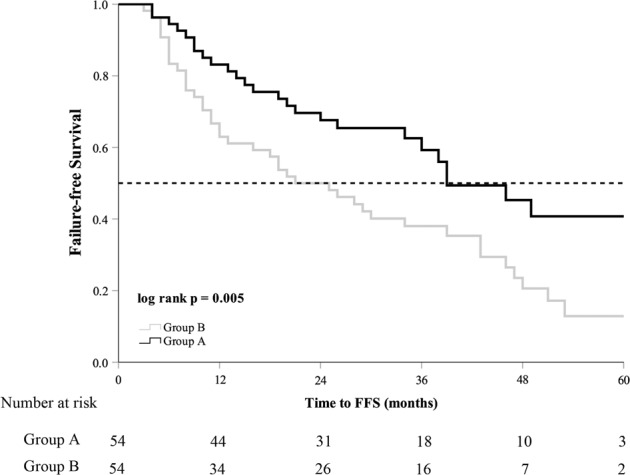
Failure-free survival in the study population. Group A cryoablation + ADT, group B ADT alone, ADT androgen deprivation therapy, FFS failure-free survival.

**Fig. 2:**
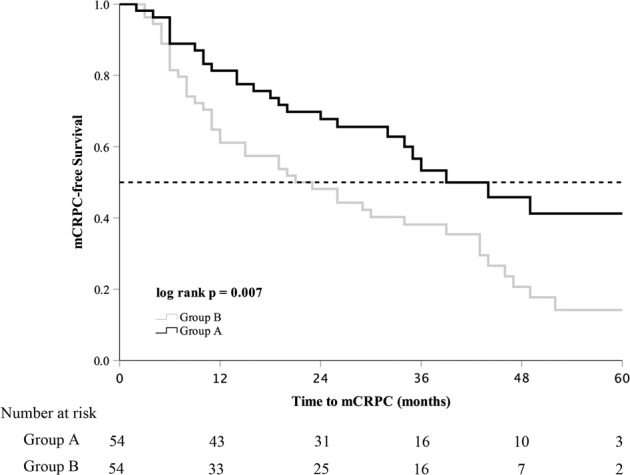
Metastatic castration-resistant prostate cancer-free survival in the study population. Group A cryoablation + ADT, group B ADT alone, ADT androgen deprivation therapy, mCRPC metastatic castration-resistant prostate cancer.

**Fig. 3:**
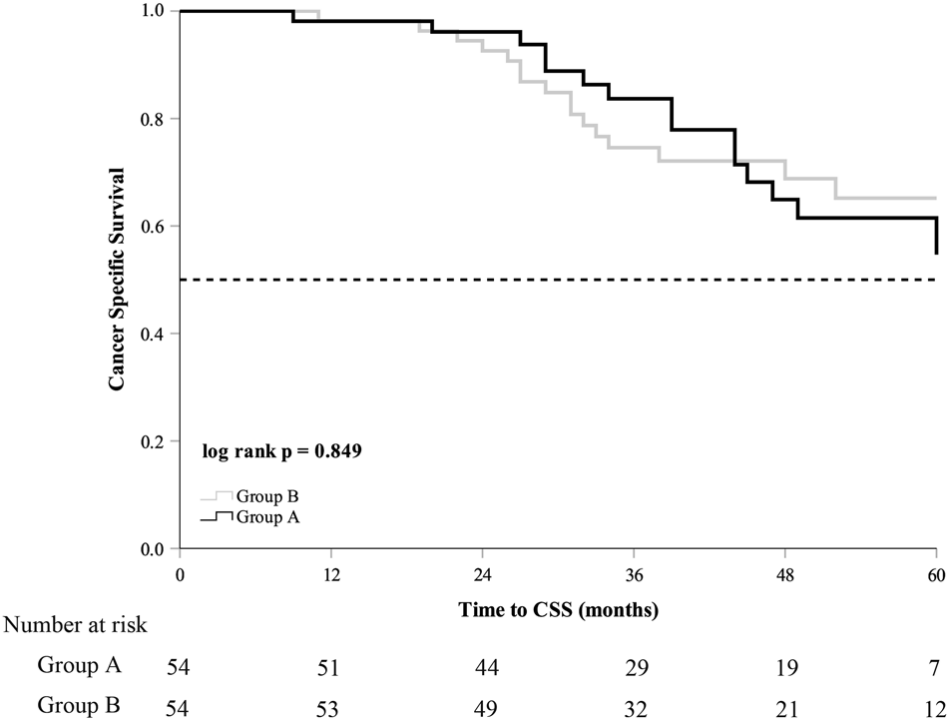
Cancer-specific survival in the study population. Group A cryoablation + ADT, group B ADT alone, ADT androgen deprivation therapy, CSS cancer-specific survival.

**Fig. 4:**
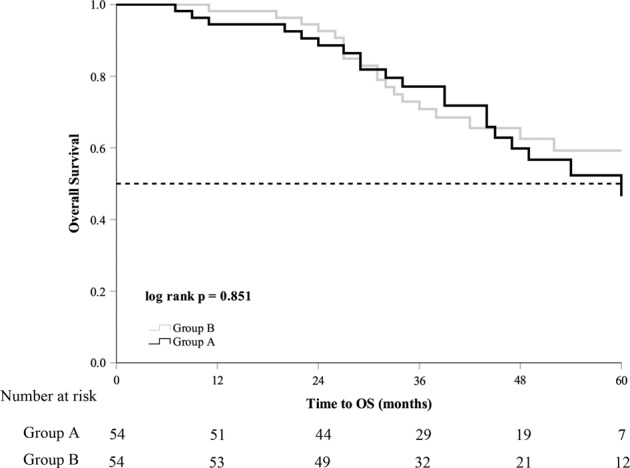
Overall survival in the study population. Group A cryoablation + ADT, group B ADT alone, ADT androgen deprivation therapy, OS overall survival.

The median follow-up time was 40 (27–53) months in group A and 39 (31–54) months in group B (*p* = 0.263). The median serum PSA nadir in group A was lower than that in group B (0.025 ng/mL vs. 0.230 ng/mL, *p* = 0.001). Until the last follow-up, 25 (46.3%) patients in group A and 42 (77.8%) patients in group B progressed to mCRPC. mCRPC patients in group A received abiraterone acetate plus prednisone (11, 44.0%), chemotherapy (3, 12.0%), estramustine phosphate (6, 24.0%), or flutamide (3, 12.0%). mCRPC patients from group B received abiraterone acetate plus prednisone (18, 42.9%), chemotherapy (14, 33.3%), estramustine phosphate (13, 40.0%), or flutamide (3, 7.1%). The patients in group A had longer FFS and mCRPC-free survival than group B (39 months vs. 21 months, *p* = 0.005; 39 months vs. 21 months, *p* = 0.007, respectively) (Table 2). The Kaplan–Meier curve showed that patients in group A had longer FFS and mCRPC-free survival (log­rank test *p* = 0.005, *p* = 0.007, Figs. 1 and 2). The univariate analysis showed that FFS was significantly associated with T stage, metastatic burden, and additional CA (*p* = 0.006, *p* = 0.046, *p* = 0.007, respectively). After adjusting for the effects of these parameters in the multivariate analysis, CA was an independent predictor of FFS (hazard ratio, 0.542; 95% confidence interval, 0.329–0.893; *p* = 0.016) (Table 3). There was no significant difference in CSS and OS between the two groups (Figs. 3 and 4).

**Table 2:** Oncological results in the study cohort according to the degree of metastasis.

	Group A	Group B	*p* value
Median follow-up (mo)	40 (27–53)	39 (31–54)	0.263
All patients	54	54	
Median PSA nadir, ng/mL	0.025 (0.003–0.273)	0.230 (0.051–1.035)	0.001
Median FFS (mo)	39.0 ± 5.79	21.0 ± 5.13	0.005
Median time to mCRPC (mo)	39.0 ± 7.22	21.0 ± 5.71	0.007
Median CSS (mo)	NR	75.0 ± 6.50	0.849
Median OS (mo)	NR	67.0 ± 12.18	0.851
Deaths from PCa	15 (27.8%)	20 (37.0%)	0.304
Deaths from all causes	20 (37.0%)	23 (42.6%)	0.555
Low metastatic burden patients	25	24	
Median PSA nadir, ng/mL	0.030 (0.003–0.222)	0.173 (0.041–1.001)	0.037
Median FFS (mo)	NR	28.0 ± 15.90	0.190
Time to mCRPC(mo)	NR	26.0 ± 14.98	0.195
Median CSS (mo)	60.0 ± 10.00	NR	0.280
Median OS (mo)	54.0 ± 5.72	NR	0.144
Deaths from PCa	7 (28.0%)	4 (16.7%)	0.342
Deaths from all causes	10 (40.0%)	5 (20.8%)	0.146
High metastatic burden patients	29	30	
Median PSA nadir, ng/mL	0.020 (0.003–0.459)	0.258 (0.079–1.035)	0.012
Median FFS (mo)	46.0 ± 10.24	18.0 ± 6.16	0.010
Time to mCRPC(mo)	44.0 ± 8.53	15.0 ± 6.16	0.011
Median CSS (mo)	NR	66.0 ± 9.78	0.434
Median OS (mo)	NR	52.0 ± 14.98	0.488
Deaths from PCa	8 (27.6%)	16 (53.3%)	0.044
Deaths from all causes	10 (34.5%)	18 (60.0%)	0.050

Group A, cryoablation + ADT. Group B, ADT alone.

*ADT* androgen deprivation therapy, *mo* months, *PSA* prostate-specific antigen, *FFS* failure-free survival, *mCRPC* metastatic castration-resistant prostate cancer, *CSS* cancer-specific survival, *OS* overall survival, *PCa* prostate cancer, *NR* not reached.

**Table 3:** Cox proportional hazards analysis of failure-free survival in the study population.

Variables	Univariate analysis		Multivariate analysis	
	HR (95% CI)	*p* value	HR (95% CI)	*p* value
Age (years)	0.984 (0.956–1.012)	0.255	–	–
ECOG PS		0.603		–
0	1		–	
>0	1.188 (0.621–2.269)		–	
PSA (ng/mL)	1.001 (1.000–1.001)	0.054	1 (1.000–1.001)	0.443
Gleason score		0.068		0.193
<8	1		1	
≥8	1.597 (0.965–2.642)		1.411 (0.840–2.369)	
T Stage		0.006		0.026
<3	1		1	
≥3	2.127 (1.243–3.637)		1.881 (1.078–3.283)	
N Stage		0.288		0.859
0	1		1	
1	1.300 (0.801–2.112)		1.048 (0.626–1.753)	
Visceral metastases		0.945		0.463
0	1		1	
1	1.025 (0.507–2.027)		0.757 (0.359–1.594)	
Metastatic burden		0.046		0.046
Low	1		1	
High	1.657 (1.009–2.722)		1.712 (1.010–2.903)	
Cryoablation	0.505 (0.308–0.830)	0.007	0.542 (0.329–0.893)	0.016

*ECOG PS* Eastern Cooperative Oncology Group Performance Status, *PSA* prostate-specific antigen, *HR* hazard ratio, *CI* confidence interval.

Among patients with low metastatic burden, PSA nadir was lower in group A than in group B (0.030 ng/mL vs. 0.173 ng/mL, *p* = 0.037). Among patients with high metastatic burden, group A had lower median PSA nadir, longer median FFS and median time to mCRPC, and decreased mortality from PCa than group B (*p* = 0.012, *p* = 0.010, *p* = 0.011, and *p* = 0.044, respectively) (Table 2).

### Symptom control and primary lesion treatment

CA was well tolerated in 54 mPCa patients within 6 months of ADT. No patients died during surgery, and no patients presented urethrorectal fistula or urinary incontinence. The complications were shown in Table 4 according to Clavien–Dindo Classification. Nonetheless, these complications disappeared within 4 weeks after surgery. Forty-three patients (79.6%) in group A and 44 patients (81.5%) in group B had urinary symptoms at diagnosis, respectively. Patients in group A had greater relief of urinary symptoms at the mHSPC stage (79.1 vs. 59.1%, *p* = 0.044, Table 5). At the mCRPC stage, complications due to primary lesion progression occurred in eight (32.0%) patients in group A and 18 (42.9%) patients in group B (*p* = 0.378, Table 5 Supplementary Table 1). Seven (13.0%) patients in group A and 17 (31.5%) in the control group received local treatment of primary lesions for symptomatic relief (*p* = 0.021). The chosen treatments were transurethral resection of the prostate (TURP) (one patient, 1.9%), urethral dilation (five patients, 9.3%), or radiotherapy (one patient, 1.9%) in group A, and TURP (two patients, 3.7%), suprapubic cystostomy (one patient, 1.9%), catheterization (two patients, 3.7%), or radiotherapy (12 patients, 22.2%) in group B.

**Table 4:** Cryoablation-related complications stratified by Clavien–Dindo classification.

Complications	No. of patients
Grade I	13 (24.1%)
Scrotal edema	8 (14.8%)
Hematuria	3 (5.6%)
Urinary retention	2 (3.7%)
Grade II	0
Grade III	0
Grade IV	0

**Table 5:** Urinary symptoms at the metastatic hormone-sensitive prostate cancer stage and complications due to primary lesion progression at the metastatic castration-resistant prostate cancer stage in the study population.

	Group A	Group B	*p* value
Urinary symptoms at diagnosis	43 (79.6%)	44 (81.5%)	0.808
Relief of urinary symptoms at the mHSPC stage	34 (79.1%)	26 (59.1%)	0.044
Complications due to primary lesion progression at the mCRPC stage	8 (32.0%)	18 (42.9%)	0.378
Local symptomatic treatment	7 (13.0%)	17 (31.5%)	0.021

*Group A* cryoablation + ADT, *group B* ADT alone, *ADT* androgen deprivation therapy, *mCRPC* metastatic castration-resistant prostate cancer, *mHSPC* metastatic hormone-sensitive prostate cancer.

## Discussion

In this retrospective study, PSM was applied to determine the oncological and clinical efficacy of additional CA with ADT for treating newly diagnosed mPCa. The results showed that combination therapy reduced serum PSA nadir, prolonged FFS and mCRPC-free survival, relieved urinary symptoms at the mHSPC stage, and reduced the need for the palliative treatment of primary tumors.

Although ADT is one of standard therapies for mPCa, recent studies have shown that mPCa patients may benefit from the local treatment of PCa. Two randomized trials [9, 10] showed that additional radiotherapy increased survival in low metastatic burden patients. Leyh-Bannurah et al. [15] used SEER data to demonstrate that radical prostatectomy improved CSS, especially in M1a patients. Heidenreich et al. [8] performed a case-control study and showed that CRP plus ADT prolonged PFS and OS in low metastatic burden patients; however, only 23 patients were included in the operation group, and their PSA levels were lower than those in the control group before treatment (*p* = 0.049).

Few studies have evaluated the clinical efficacy and oncological outcomes of CA in mPCa, although this treatment is minimally invasive and effective. Si et al. [16] found that CA plus ADT prolonged PFS, CSS, and OS in mPCa patients (*p* < 0.01). However, only 30 patients were included in the CA group, and the patients in the ADT group were selected by pair-matched analysis, which might have caused selection bias. Our analysis used PSM to avoid this type of bias, and the results showed that CA plus ADT prolonged FFS and mCRPC-free survival, and achieved lower median PSA nadir in mPCa patients. Some studies [17–19] have shown that the PSA nadir is a powerful predictor of OS in mPCa patients receiving ADT.

Additional CA in mPCa patients might achieve antitumor effects by delaying cancer metastasis and improving immunity. Chu et al. [20] demonstrated that intact tumor-specific antigens produced by tumor CA in situ could trigger an immune response and help fight cancer. Benzon et al. [21] analyzed the curative effect of CA and immune therapy in a mouse model and found that combination treatment postponed the growth of metastatic tumors (*p* = 0.0006). Connor et al. [22] found that minimally invasive ablative therapies, including CA, killed tumor cells and induced cellular and humoral antitumor immune responses by releasing tumor-associated antigens. Our results showed that CA plus ADT prolonged FFS and mCRPC-free survival, and reduced mortality in high metastatic burden patients receiving CA (Table 2). We hypothesized that systemic antitumor immunity activated by CA played a role, although Kaplan–Meier and log-rank analyses showed that there were no significant intergroup differences in CSS. The small sample size may have caused bias, and larger studies are necessary to evaluate patients with high metastatic burden. These outcomes support that killing cells from the primary tumor may prevent metastasis.

ADT may not completely relieve urinary symptoms in mPCa patients, and primary lesion progression may cause complications. Choi et al. [23] analyzed International Prostate Symptom Score (IPSS) voiding symptoms in 110 PCa patients treated with ADT and found that ADT improved the IPSS, but the curative effect decreased after 1 year of treatment (–4.10 within 1 year vs. –2.65, *p* < 0.05). Akpayak et al. [24] carried out a prospective study and showed that 12 months after ADT, the IPSS decreased in 50.8% of mPCa patients with moderate or severe urinary symptoms. Furthermore, urinary symptoms may have worsened in patients in which PCa progressed to mCRPC. Won et al. [6] showed that 54.3% of patients who underwent ADT alone had complaints at the CRPC stage, and 20% required further palliative external beam radiotherapy because of local prostatic symptoms. Several studies showed that cytoreductive surgery for primary tumors in mPCa patients could control local disease. Steuber et al. [25] conducted a case-control study and demonstrated that mPCa patients undergoing CRP had lower severe local complications compared with the control group without CRP (7.0 vs 35%; *p* < 0.01). Heidenreich et al. [8] found that CRP combined with ADT reduced the risk of local complications in mPCa patients. In the present study, patients in the CA group had higher relief of urinary symptoms than the control group (Table 5). Moreover, fewer mPCa patients in the CA group had complications at the mCRPC stage, although the intergroup difference was not significant (32.0 vs. 42.9%, *p* = 0.378). The most common complication after mCRPC in our cohort was BOO, which is in accordance with the results of Won et al. [6]. In addition, the CA group required less palliative treatment of primary lesions for symptomatic relief compared to the control group (13.0 vs. 31.5%, *p* = 0.021). Compared with CRP, CA was less invasive for mPCa patients, who were predominantly elderly people; nonetheless, the oncological outcomes of localized PCa were similar between CA and radical prostatectomy [26, 27].

This study has some limitations. First, the retrospective nature of the study may have led to bias. Second, the follow-up period was not long enough to achieve median CSS and OS in the CA group. Third, the number of cases was small. Notwithstanding, a prospective randomized control clinical trial is in progress in our hospital to address these limitations.

## Conclusions

Our results suggest that prostate CA combined with ADT for newly diagnosed mPCa patients improves oncologic outcomes with longer FFS and mCRPC-free survival, and lower PSA nadir. Moreover, this combination treatment can reduce urinary symptoms and decrease the need for treating primary lesions for symptomatic relief. Notwithstanding, further prospective studies are needed to confirm these conclusions.

## Supplementary information

Supplementary Table 1
