# Supplementary material for: Prostate cryoablation combined with androgen deprivation therapy for newly diagnosed metastatic prostate cancer: a propensity score-based study
Source: Prostate Cancer Prostatic Dis. 2021 Mar 4;24(3):837–44. doi: 10.1038/s41391-021-00335-2 (PMC8384623; doi:10.1038/s41391-021-00335-2)
Supplement: Supplementary file 1 — Supplementary Table 1 [file 41391_2021_335_MOESM1_ESM.docx]

|  | Complications | Hematuria | Bladder outlet obstruction | Acute urinary retention | Ureteric  obstruction |
| --- | --- | --- | --- | --- | --- |
| Group A | 8 (32.0%) | 4 (16.0%) | 7 (28.0%) | 0 | 0 |
| Group B | 18 (42.9%) | 1 (2.4%) | 18 (42.9%) | 4 (9.5%) | 0 |
| Total | 26 (38.8%) | 5 (7.5%) | 25 (37.3%) | 4 (6.0%) | 0 |

Supplementary Table 1 Complications due to primary lesion progression at the metastatic castration-resistant prostate cancer stage in the study cohort.

Group A, cryoablation + ADT; group B, ADT alone; ADT, androgen deprivation therapy.
